# Maternal Metformin Intervention during Obese Glucose-Intolerant Pregnancy Affects Adiposity in Young Adult Mouse Offspring in a Sex-Specific Manner

**DOI:** 10.3390/ijms22158104

**Published:** 2021-07-28

**Authors:** Josca M. Schoonejans, Heather L. Blackmore, Thomas J. Ashmore, Catherine E. Aiken, Denise S. Fernandez-Twinn, Susan E. Ozanne

**Affiliations:** 1 University of Cambridge Metabolic Research Laboratories and MRC Metabolic Diseases Unit, Wellcome-MRC Institute of Metabolic Science, University of Cambridge, Addenbrooke’s Treatment Centre, Keith Day Road, Cambridge CB2 0QQ, UK; hlb46@medschl.cam.ac.uk (H.L.B.); tja34@medschl.cam.ac.uk (T.J.A.); df220@cam.ac.uk (D.S.F.-T.); 2 Department of Obstetrics and Gynaecology, University of Cambridge, Cambridge CB2 0SW, UK; cema2@cam.ac.uk

**Keywords:** gestational diabetes, foetal programming, metformin, maternal obesity, white adipose tissue, sex differences

## Abstract

Background: Metformin is commonly used to treat gestational diabetes mellitus. This study investigated the effect of maternal metformin intervention during obese glucose-intolerant pregnancy on the gonadal white adipose tissue (WAT) of 8-week-old male and female mouse offspring. Methods: C57BL/6J female mice were provided with a control (Con) or obesogenic diet (Ob) to induce pre-conception obesity. Half the obese dams were treated orally with 300 mg/kg/d of metformin (Ob-Met) during pregnancy. Gonadal WAT depots from 8-week-old offspring were investigated for adipocyte size, macrophage infiltration and mRNA expression of pro-inflammatory genes using RT-PCR. Results: Gestational metformin attenuated the adiposity in obese dams and increased the gestation length without correcting the offspring in utero growth restriction and catch-up growth caused by maternal obesity. Despite similar body weight, the Ob and Ob-Met offspring of both sexes showed adipocyte hypertrophy in young adulthood. Male Ob-Met offspring had increased WAT depot weight (*p* < 0.05), exaggerated adipocyte hyperplasia (*p* < 0.05 vs. Con and Ob offspring), increased macrophage infiltration measured via histology (*p* < 0.05) and the mRNA expression of *F4/80* (*p* < 0.05). These changes were not observed in female Ob-Met offspring. Conclusions: Maternal metformin intervention during obese pregnancy causes excessive adiposity, adipocyte hyperplasia and WAT inflammation in male offspring, highlighting sex-specific effects of prenatal metformin exposure on offspring WAT.

## 1. Introduction

The prevalence of obesity is rising at sufficiently rapid rates such that over 50% of women of child-bearing age in the UK are currently overweight or obese [1]. Obesity is not only associated with adverse metabolic and cardiovascular events in the adult female [2], but, when present during pregnancy, it also has long-term ‘programming effects’ on exposed offspring that increase their risk of obesity, insulin resistance, type 2 diabetes and the metabolic syndrome [3].

Women with obesity are four times more likely to develop gestational diabetes mellitus (GDM) during pregnancy [4]. Metformin is the first-line pharmacological treatment for GDM in many countries, including the UK, when lifestyle interventions are ineffective, and its use has been increasing worldwide [5,6]. In pregnancy, metformin improves glucose tolerance in women with GDM, and metformin treatment is associated with a lower gestational weight gain compared to insulin or placebo in GDM or obese glucose-tolerant women, respectively [7,8]. Its oral administration, lack of requirement for refrigerated storage, and cost-effectiveness also make it more appropriate than insulin for use in low-resource settings. Therefore, it is plausible that metformin use in pregnancy might offer a suitable intervention to mitigate or negate the adverse effects of an obese diabetic intrauterine environment on offspring health globally. However, metformin readily crosses the placenta [9], and offspring follow-up in human randomised controlled trials investigating maternal metformin treatment remains sparse, with few studies reporting offspring outcomes beyond infancy [10,11,12,13,14,15]. However, the fact that several studies have reported an increased adiposity in children exposed to maternal metformin treatment in pregnancy warrants further investigation into the long-term effects [10,12,13]. Animal studies are therefore essential to determine the direct and indirect effects of maternal and in utero metformin exposure on offspring adiposity, adipose tissue biology and metabolic health. Moreover, animal studies allow the investigation of potentially sexually dimorphic effects, whereas human follow-up studies are often insufficiently powered to perform this analysis.

In our well-established mouse model of maternal diet-induced obesity [16], dams are fed an obesogenic diet high in both fat and sugar before mating and are consequently obese, hyperleptinaemic and hyperinsulinaemic at conception and develop glucose intolerance in pregnancy [16,17,18]. It was previously found that male offspring of obese dams develop obesity from 12 weeks of age, while adipocyte hypertrophy is present from as early as 8 weeks of age [16,19,20]. We and others have shown that the programming effects of maternal obesity on male rodent offspring adiposity and adipose tissue function can be ameliorated by maternal exercise intervention [17,21,22]. Although the effects of early life exposure to metformin on offspring body composition have been reported in several rodent models [23,24,25,26,27,28,29], the effects of clinically relevant maternal metformin intervention exclusively during obese glucose-intolerant pregnancy (and not lactation) on offspring WAT have not been studied. Furthermore, growing evidence indicates that programming effects can be sexually dimorphic, with males and females responding differently or showing a different time course of response to a suboptimal in utero environment [30], highlighting the importance of including both male and female offspring in this study.

This study therefore aimed to investigate the effect of maternal metformin intervention during obese glucose-intolerant pregnancy on 8-week-old offspring gonadal WAT using a mouse model of diet-induced obesity. Importantly, as it has emerged that there can be sex-specific effects of in utero exposures, this study included both male and female offspring. We report that maternal obesity caused gonadal adipocyte hypertrophy, which was not corrected by the metformin intervention. Instead, the metformin intervention increased adipocyte hyperplasia, macrophage infiltration and gonadal WAT inflammation in male but not female offspring. 

## 2. Results

### 2.1. Metformin Attenuates Fat Mass in Diet-Induced Obese Dams

Ob and Ob-Met dams were fed an obesogenic diet prior to mating, and hence had a higher body weight and fat mass than Con dams did in late gestation (Table 1). The metformin intervention decreased maternal body weight and fat mass (but not lean mass) at E15.5. Maternal obesity caused a reduction in litter size (from 7 to 6 pups per litter, Table 1). This reduction was not prevented by maternal metformin treatment (Table 1). Maternal obesity also led to a decreased pup weight on postnatal day 2 (PN2) (from 1.9 to 1.6 g per pup, Table 1). This growth restriction was not prevented by maternal metformin administration (Table 1), despite the increased gestation length observed in metformin-treated obese pregnancies (Table 1).

### 2.2. Maternal Metformin Treatment Induces Adiposity in Male but Not Female Offspring

**Figure 1:**
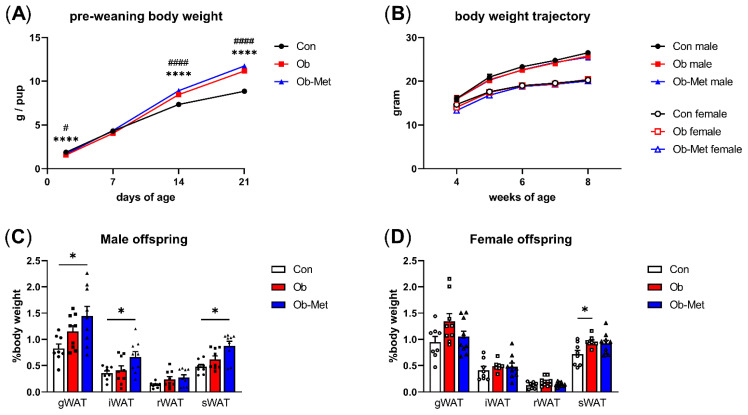
Offspring growth and adiposity in 8-week-old offspring. (**A**) Growth trajectory of neonatal offspring (n = 26–33 independent litters per group, sexes combined). **** *p* < 0.0001 vs. Con vs. Ob, ^#^ *p* < 0.05, ^####^ *p* < 0.001 Con vs. Ob-Met using two-way ANOVA with Tukey’s multiple comparison test. (**B**) Post-weaning body weight trajectory in male and female offspring until 8 weeks of age (n = 10–14 independent litters per group). (**C**,**D**) Weight of white adipose tissue (WAT) depots collected at 8 weeks of age relative to offspring body weight (n = 8–10 independent litters per group). Outliers were excluded from (**C**) iWAT and sWAT; female Ob (Grubb’s method, outlier excluded values 1.03% and 1.45%, respectively). * *p* < 0.05 using one-way ANOVA with Tukey’s multiple comparison test. gWAT = gonadal depot, iWAT = intraperitoneal depot, rWAT = retroperitoneal depot, sWAT = subcutaneous depot. Con (circles, solid line) = offspring of control-fed dams, Ob (squares, dashed line) = offspring of obese dams, Ob-Met (triangles, dotted and dashed line) = offspring of obese metformin-treated dams. Panels (**B**–**D**): closed symbols denote male offspring; open symbols denote female offspring.

Pups born to Ob dams were lighter at PN2 but displayed catch-up growth leading to higher pup weights than the controls by PN14, and this difference remained at PN21. This was not corrected by the metformin intervention (Figure 1A). After weaning, there were no differences in body weight between any of the three groups up to 8 weeks of age (Figure 1B). However, the weights of the gonadal, intraperitoneal, and subcutaneous WAT depots at 8 weeks of age were significantly increased in male Ob-Met offspring (Figure 1C). This was not observed in female Ob-Met offspring (Figure 1D).

### 2.3. Metabolic Health Is Not Affected by Maternal Obesity or Metformin Exposure

Serum insulin did not significantly differ between groups in either male (Con 50 ± 6, Ob 67 ± 6, Ob-Met 73 ± 10 pmol/L, *p* = 0.1697, one-way ANOVA) or female offspring (Con 77 ± 15, Ob 67 ± 7, Ob-Met 52 ± 4 pmol/L, *p* = 0.1417, one-way ANOVA). Similarly, there was no difference in glucose tolerance, as measured by the intraperitoneal glucose tolerance test in male (Con 1340 ± 51, Ob 1448 ± 57, Ob-Met 1343 ± 67 AUC, *p* = 0.3463, one-way ANOVA) or female offspring (Con 1209 [1183–1321], Ob 1274 [1106–1379], Ob-Met 1235 [1160–1416] AUC, *p* = 0.9920, Kruskal–Wallis test).

### 2.4. Gonadal WAT Cellularity Is Altered by Maternal Obesity and Metformin Intervention

**Figure 2:**
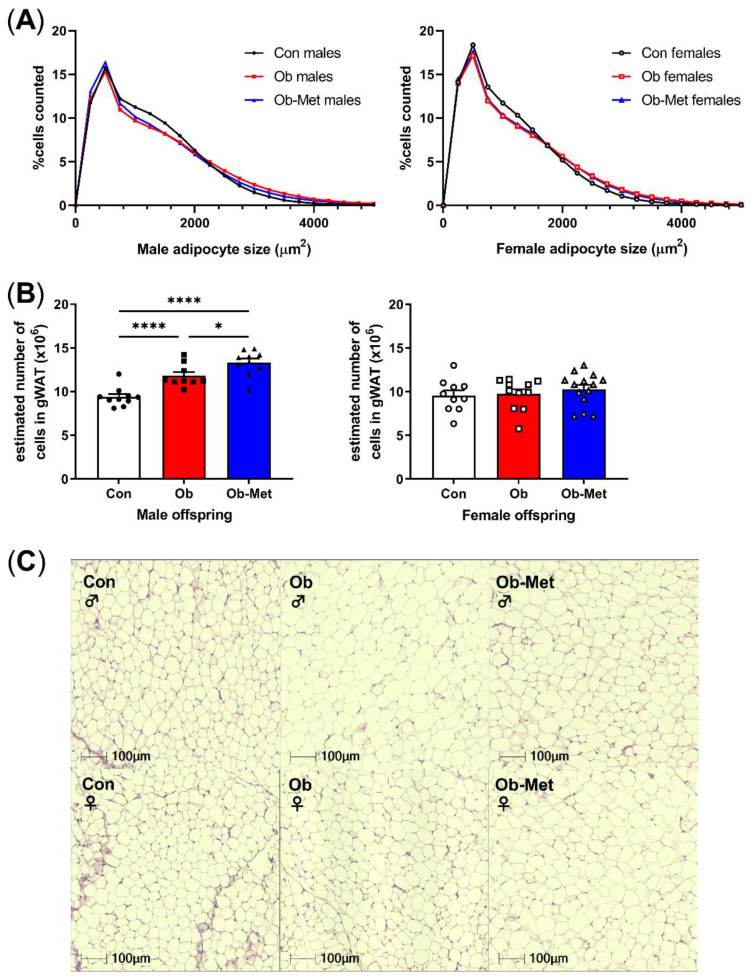
Cellularity of 8-week-old gonadal adipocytes. (**A**) Adipocyte size distribution in male (left) and female (right) 8-week-old offspring. (**B**) Estimated adipocyte number in the collected gonadal white adipose tissue (WAT) depot. * *p* < 0.05, **** *p* < 0.0001 using one-way ANOVA with Tukey’s multiple comparison test. (**C**) Representative images of H&E-stained sections used for cell size analysis. Con (circles, solid line) = offspring of control-fed dams, Ob (squares, dashed line) = offspring of obese dams, Ob-Met (triangles, dotted and dashed line) = offspring of obese metformin-treated dams. Closed symbols denote male offspring; open symbols denote female offspring. n = 9–14 independent litters per group. Outliers were excluded from (**B**); male Con (Grubb’s method, outlier excluded value 6.28 × 10^6^).

Male and female Ob and Ob-Met offspring showed gonadal adipocyte hypertrophy indicated by the rightward shift in the adipocyte size distribution compared to Con offspring (Figure 2A,C, *p* < 0.0001 for the interaction between the maternal environment and adipocyte size, two-way ANOVA). This was particularly apparent in the larger adipocyte range, although male Ob-Met offspring also showed an increase in small adipocytes (Figure 2A, left). Maternal obesity increased the estimated adipocyte number in male but not female offspring, and this was exaggerated by exposure to maternal metformin treatment with male offspring of obese metformin-treated dams having significantly more adipocytes than male offspring of obese (and control) dams did (Figure 2B).

### 2.5. Maternal Metformin Introduces Gonadal WAT Inflammation in Male Offspring

**Figure 3:**
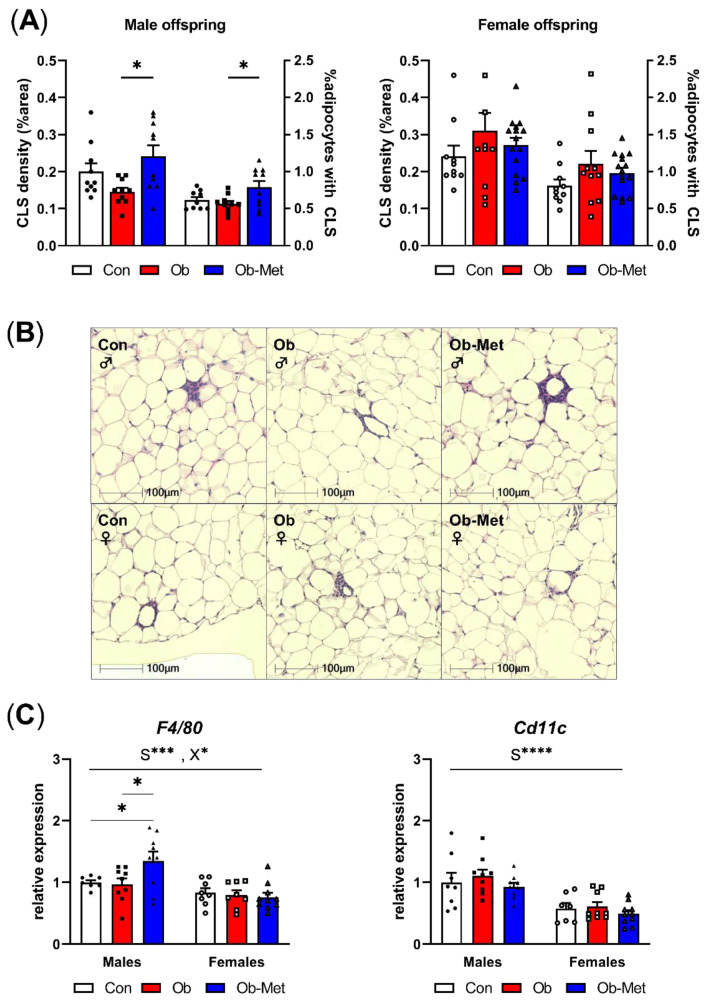
Inflammation in gonadal white adipose tissue. (**A**) Presence of crown-like structures (CLS) in gonadal white adipose tissue (WAT) of 8-week-old male (left) and female (right) offspring, expressed as percentage area of the WAT tissue and as the number of CLS divided by the number of adipocytes in the section (n = 10–15 independent litters per group). * *p* < 0.05 using one-way ANOVA with Tukey’s multiple comparison test. (**B**) Representative images of H&E-stained sections used for CLS analysis. (**C**) mRNA expression of macrophage markers in gonadal WAT relative to the expression of housekeeper gene *Ppia* and the male Con group (n = 7–10 independent litters per group). * *p* < 0.05, *** *p* < 0.001, **** *p* < 0.0001 for the main effect of sex (S), interaction between sex and the maternal environment (X) or Tukey’s multiple comparison using two-way ANOVA. Outliers were excluded from (**A**) CLS density; female Con (Grubb’s method, outlier excluded value 0.46%), and (**A**) %adipocytes with CLS; male Con (Grubb’s method, outlier excluded value 1.19%). From (**C**) *F4/80*, two data points were excluded due to pipetting errors (male Con and female Ob). From (**C**) *Cd11c*, one data point was excluded due to pipetting errors (female Con).

Male Ob-Met offspring showed increased CLS in adipose tissue, indicating a pro-inflammatory signature of the gonadal WAT (Figure 3A,B). This was not seen in female offspring. Accordingly, the expression of the macrophage marker *F4/80* was specifically increased in male Ob-Met offspring compared to both Con and Ob offspring WAT (Figure 3C). *Cd11c* (a pro-inflammatory M1-type macrophage marker) expression was not affected by maternal obesity or metformin treatment. Overall, *F4/80* and *Cd11c* expressions were lower in female compared to male WAT.

## 3. Discussion

This study showed that maternal metformin treatment during obese pregnancy decreased maternal fat mass and body weight in late gestation, but failed to correct the decreased litter size, growth restriction with catch-up growth phenotype and adipocyte hypertrophy in offspring exposed to maternal obesity. Moreover, in 8-week-old male offspring, maternal metformin intervention exaggerated the adiposity phenotype characterised by excessive adipocyte hyperplasia, macrophage infiltration and expression of pro-inflammatory genes. This was not observed in female offspring.

Metformin is known to reduce body weight in nonpregnant type 2 diabetic patients [31], and an effect of metformin to lower gestational weight gain is a common observation in human trials in GDM, polycystic ovary syndrome (PCOS) and obese women [8,32,33]. Our findings of lower body weight and fat mass, consistent with a protective effect of metformin on diet-induced obesity in late gestation, are therefore in accordance with human data, supporting the validity of our model in assessing other effects of metformin treatment during pregnancy, including the potential long-term effects of metformin exposure in utero on offspring health. As metformin can cross the placenta and enter the foetal circulation, attaining similar circulating concentrations to those in the mother, the need for long-term follow-up studies has been highlighted [34]. As these studies are both challenging to carry out and take many years in humans, animal models are important to address this question.

Maternal obesity during pregnancy is associated with an increased risk of both small and large for gestational age babies [35]. Consistent with previous studies in the model [16,36,37], we observed growth restriction followed by catch-up growth in offspring exposed to maternal obesity. The combination of low birth weight and accelerated postnatal growth is associated with long-term adverse effects on offspring cardiometabolic health in human studies [38,39,40,41] and animal models [42,43]. The metformin intervention did not correct (or worsen) this pattern of early growth. However, our study is the first to report that metformin intervention in the obese mouse dam increased gestation time by one day. Therefore, the equivalent growth restriction observed in both Ob and Ob-Met pups, despite the delay in parturition, might suggest that metformin-exposed foetuses would be further growth-restricted compared to nonexposed foetuses if assessed on the same gestational day.

The mechanism by which metformin causes adiposity in male offspring is unclear. Metformin is known to activate AMPK, causing inhibition of mTOR, decreased cell proliferation, suppression of protein synthesis and cell cycle arrest [34]. Such a mechanism acting on foetal tissue could explain the relative growth restriction observed, which is masked in terms of birthweight due to the delay in parturition. Providing metformin in obese pregnancy but not lactation may thus reflect a model of growth restriction (due to metformin) with catch-up growth (following metformin removal at birth) similar to studies in which recuperated offspring displayed rapid post-natal growth following in utero low-protein exposure [42]. This was also proposed by Salomäki et al. who drew a comparison between their model of gestational metformin treatment to chow-fed dams and developmental programming by maternal undernutrition [26]. Indeed, when looking at studies investigating male offspring adiposity in nontransgenic rodent models (including the current study), metformin was only detrimental to offspring body composition in situations where this cellular ‘starvation’ was followed by restored or increased energy balance in lactation, whereas continued metformin treatment or switching to a healthy diet postnatally was associated with protective effects [23,25,26]. Whether this relates to direct effects of metformin signalling on foetal development or indirectly through effects on maternal metabolism is unclear. Combined with the increased gestation length in this study and the human data summarised above, this suggests that metformin may adversely affect intrauterine development, particularly for male offspring.

At 8 weeks of age, the body weight and weight of both visceral (epididymal, intraperitoneal and retroperitoneal) and subcutaneous WAT depots did not differ between Con and Ob offspring despite evidence of gonadal adipocyte hypertrophy in both sexes, in accordance with previous reports in male offspring [19]. In contrast, maternal metformin treatment introduced male-specific increased adiposity, while female offspring were unaffected. The increased adiposity in male Ob-Met compared to Ob offspring was likely driven by hyperplasia as adipocyte size was unaffected by the metformin intervention. This early life hypercellularity may predispose offspring to becoming obese later in life by increasing the lifelong lipid-storage capacity of WAT. In addition to being increased in mass, the hypertrophic gonadal WAT of males exposed to metformin in utero displayed increased CLS density and mRNA expression of the macrophage marker *F4/80*, indicative of enhanced macrophage infiltration. This was not seen in Ob offspring, indicating a more progressive WAT dysfunction phenotype in Ob-Met offspring despite similar levels of hypertrophy, a known stimulus for macrophage invasion [44]. The percentage of adipocytes surrounded by CLS, a marker of adipocyte death [44,45], was also increased in Ob-Met compared to Ob offspring. This suggests that in utero metformin exposure increased the adipocyte death rate in male Ob-Met WAT, leading to the recruitment of immune cells, phagocytosis of necrotic adipocytes and production of pro-inflammatory mediators. The increased prevalence of CLS in Ob-Met gonadal WAT may be related to the increased hyperplasia as ‘obesity-induced adipocyte death’ has been suggested as the limiting factor to adipocyte hypertrophy and may underlie the switch from hypertrophic to hyperplastic adiposity [44]. Inflammation is a major contributor to adipose tissue insulin resistance. However, no adverse metabolic phenotype was observed in Ob-Met offspring at this age. This is not surprising, as WAT inflammation is known to precede systemic hyperinsulinaemia [46], suggesting that metabolic alterations may develop at a later age.

Female offspring were relatively protected against programmed adiposity in this study with no effects of either maternal obesity or metformin on weights of the visceral WAT depots. Female 8-week-old offspring also showed a lower expression of pro-inflammatory genes compared to males, consistent with reports that basal cytokine and chemokine expressions are higher in male compared to female WAT [47]. In contrast to males, there was no maternal environment effect on CLS formation in female offspring, indicating a sexually dimorphic susceptibility to WAT inflammation in a programming context. This is in accordance with previous reports showing that maternal high-fat feeding in pregnancy leads to macrophage invasion in chow-fed male offspring, while female offspring required a postnatal high-fat diet to show upregulated pro-inflammatory gene expression. Even then, the phenotype was milder than that in high-fat-diet-fed males [48]. The mechanisms underlying the sex differences in our study are unknown, but anti-inflammatory actions of oestrogen in female offspring are suggested as one explanation [48]. Sexual dimorphism in a developmental programming context is becoming increasingly recognised, with male offspring often more vulnerable to suboptimal in utero exposures than female offspring are, and subsequently displaying earlier and/or more severe phenotypes in adulthood. This may be related to sexually dimorphic patterns and the timing of development, different hormonal intrauterine and postnatal environments and the observation that males grow faster in utero than females do [49,50].

The murine data we report here are broadly consistent with the limited data available in humans. We showed in a recent meta-analysis of human randomised controlled trials that metformin treatment in gestational diabetic pregnancies is associated with decreased birth weight compared to those treated with insulin [51]. Although usually interpreted as beneficial (prevention of macrosomia), this may reflect the relative growth restriction that is masked by the obesogenic glucose-intolerant environment. Accordingly, we showed that the decreased birth weight observed in metformin-treated human GDM pregnancies is followed by increased postnatal growth, leading to heavier infants compared to insulin-treated GDM [51]. The longest human follow-up of metformin-treated GDM pregnancies to date is from the MiG trial in Australasia. In 2011, this trial reported an increased adiposity in two-year-old metformin-exposed offspring. As no detectable change was seen in visceral fat mass at this age, the authors proposed that this might reflect expansion of the more metabolically beneficial subcutaneous depot [52]. Our current animal study, however, does not support depot-specificity but instead shows a global adiposity phenotype involving both subcutaneous and visceral depots, the latter of which was also shown by another mouse study [26]. Therefore, it is interesting to note that further follow-up from the MiG trial has since shown that adiposity in 9-year-old offspring was indeed globally increased [10]. Furthermore, our meta-analysis of GDM intervention trials also confirmed that compared to insulin, the lower birth weight and accelerated postnatal growth in metformin-exposed babies lead to childhood adiposity [51], consistent with the current mouse study.

Similar to findings in a GDM context, a placebo-controlled trial in PCOS women showed excessive postnatal growth in metformin-exposed offspring with body weight and BMI diverging from the placebo arm from 6 months of age [12]. Moreover, further follow-up of offspring born to metformin-treated PCOS women up to 10 years of age showed increased overweight and obesity [12,13], with an increased proportion of metabolically abnormal obese children in the metformin arm. In contrast, trials in obese glucose-tolerant women failed to show a relationship between metformin exposure and offspring adiposity at least up to 4 years of age [8,14,53], highlighting the importance of the longer-term follow-up of metformin-exposed offspring.

In addition to the different clinical indications, it is important to note that the timing of metformin administration differs between women treated for GDM (in the second or third trimester) and those with PCOS or type 2 diabetes (before or at conception) or studies in nondiabetic obese women (end of the first trimester). However, the common outcome of increased offspring adiposity in the context of PCOS and GDM suggests that exposure during the last trimester is critical in relation to effects on adiposity. Unfortunately, most human studies are not sufficiently powered to analyse potential sexual dimorphism in offspring outcomes. The paucity of long-term offspring follow-up in humans means that more evidence is required to determine potential critical time windows of exposure, sexual dimorphism in foetal responses, as well as the interaction with maternal metabolic status.

In conclusion, maternal metformin treatment during obese pregnancy decreased maternal fat mass in late gestation, providing a less obese intrauterine environment for developing foetuses, and consistent with beneficial effects on maternal physiology. However, maternal metformin treatment did not prevent foetal growth restriction, despite an increase in gestation length, or the postnatal catch-up growth phenotype in offspring of obese dams. These findings suggest that beneficial effects in maternal physiology do not necessarily translate to improved physiology in the offspring. Furthermore, we observed sex-specific effects of maternal metformin on offspring adiposity, with exposed males demonstrating increased adiposity and adipose tissue inflammation that were not observed in the females. These findings highlight the presence of sexually dimorphic responses to in utero exposures and the need to consider immediate, as well as postnatal, effects of treatments during pregnancy on mother and child. Given the importance of ageing in exaggerating programmed phenotypes and in the development of metabolic disease pathology, it is imperative that these offspring are followed up to determine whether these alterations in adipose biology in young adult life become pathological in later life.

## 4. Materials and Methods

### 4.1. Animal Model

All mouse work was performed according to the UK Home Office Animals (Scientific Procedures) Act 1986 Amendment Regulations 2012 and the University of Cambridge Animal and Welfare Ethical Review Board, approved under project licence P5FDF0206, 23 March 2017. As previously described [17], female C57BL/6J mice were randomly assigned to either a standard laboratory chow (RM1, 7% sugars, 3% fat) or a high-fat diet (10% sugars, 20% fat) supplemented with sweetened condensed milk (55% sugar, 8% fat) and vitamin and mineral pre-mix (AIN-93G-MX, Special Diets Services, Witham, UK) from weaning. After 6 weeks, they were mated for a primary pregnancy to ensure breeding effectiveness. Obese dams were mated for the experimental pregnancy when they reached a critical threshold of 12 g absolute fat mass, as measured by noninvasive time-domain nuclear magnetic resonance (TD-NMR, Bruker Minispec LF series, Bruker Optik GmbG, Essex, Germany), whereas control dams remained below 5 g absolute fat mass at mating. Dams were fed their respective diets ad libitum throughout pregnancy and lactation. Half of obese dams were treated orally with 300 mg/kg/d of metformin-hydrochloride supplemented in the condensed milk from one week pre-mating until embryonic day 19 (E18.5). This clinically relevant dose has previously been used in murine models and equates to a dose similar to those provided to pregnant women [26]. The body composition of dams in all three groups was measured by TD-NMR on E15.5. Litter size was standardised to six pups per litter on PN2. Male and female offspring were weaned onto RM1 at PN21 and housed in littermate pairs of the same sex until culling at 8 weeks of age using rising CO_2_ concentration. Excised adipose tissues from fed animals or following a 16 h fast were formalin-fixed or snap-frozen on dry ice, respectively.

### 4.2. Gene Expression

Gonadal adipose tissue was lysed on ice in QIAzol Lysis Reagent (Qiagen, Hilden, Germany) using the TissueRuptor II (Qiagen). RNA was extracted using the miRNeasy MiniKit (Qiagen) with DNase digestion (RNase-Free DNase Set, Qiagen) according to the manufacturer’s protocols. RNA concentration was determined using a NanoDrop 1000 Spectrophotometer (ThermoScientific, UK) and RNA quality was assessed using gel electrophoresis. cDNA was generated using the HiCapacity RT kit (Applied Biosystems, USA) with addition of the RNAse inhibitor (Applied Biosystems) using the Veriti™ 96-Well Thermal Cycler (Applied Biosystems). Real-time quantitative PCR was performed in duplicate using a SYBR™ Select Master Mix (Applied Biosystems) and the QuantStudio™ 7 Flex Real-Time PCR System (Applied Biosystems). Data were normalised using the comparative CT method against the expression of *Ppia* (which was unaffected by experimental group or sex) relative to the expression in the male control group. Primer sequences are shown in Table 2.

### 4.3. Histological Analysis

Formalin-fixed gonadal adipose tissue was processed, embedded and sectioned, and 3 μm sections were stained with haematoxylin and eosin (H&E). Whole sections were scanned using the Axioscan digital slide scanner (Zeiss, Germany). Images were analysed using HALO analysis software (Indica labs, USA) using DenseNet AI Plugin classifiers (‘crown-like structures’ or CLS analysis) and the Vacuole Quantification tool (adipocyte cell size) adapted to the H&E-stained WAT sections. The estimated adipocyte number was calculated based on methods previously described [28] by dividing the total volume of gonadal WAT (estimated from the tissue weight and density of rodent visceral WAT) by the mean adipocyte volume derived from the cross-sectional area measured by HALO. The percentage adipocytes surrounded by CLS, a marker of adipocyte death [44], was calculated by normalising the number of CLS by the number of adipocytes per section.

### 4.4. Intraperitoneal Glucose Tolerance Test

Following an overnight fast, offspring were injected intraperitoneally with 1 g/kg of glucose solution. Blood glucose was measured from the tail vein at baseline and 15, 30, 60 and 120 min post-injection (AlphaTRAK2, Zoetis, Parsippany-Troy Hills, NJ, USA). The area under the glucose excursion curve during the glucose tolerance test was computed using the trapezoid rule in Prism 8.0 (GraphPad, La Jolla, CA, USA).

### 4.5. Serum Analysis

Terminal blood was collected following a 16 h fast by cardiac puncture. Serum was collected after centrifugation at 3000× *g* and stored at −80 °C. Serum insulin was measured using the Ultra-Sensitive Mouse Insulin ELISA Kit (Crystal Chem, Elk Grove Village, IL, USA).

### 4.6. Statistical Analysis

Data are presented as means ± SEM and were analysed using Prism 8.0 software (GraphPad) using one-way ANOVA, two-way ANOVA or nonparametric alternatives where appropriate. Outliers were determined by the Grubb’s method and excluded as described in the figure legends. A *p*-value of *p* < 0.05 was considered statistically significant. In all cases, n refers to the number of independent litters represented.

## Figures and Tables

**Table 1:** Dam body composition and pregnancy outcomes. * *p* < 0.05, ** *p* < 0.01, *** *p* < 0.001, **** *p* < 0.0001 vs. Con, ^#^ *p* < 0.05, ^##^ *p* < 0.01, ^###^ *p* < 0.001 vs. Ob using one-way ANOVA with Tukey’s multiple comparison test, Kruskal–Wallis test for nonparametric data (gestation length and litter size) or ANOVA performed on log-transformed data (PN2 weight). E15.5 = embryonic day 15.5. ns = not significant. PN2 = postnatal day 2.

	Con	Ob	Ob-Met	*p*-Value
Dams at E15.5	*n* = 15	*n* = 13	*n* = 6–7	
Body weight (g)	36.0 ± 0.7	49.0 ± 0.8 ***	44.9 ± 1.2 ***^,#^	<0.0001
Lean mass (g)	23.9 ± 0.7	24.7 ± 0.6	23.9 ± 0.6	ns
Fat mass (g)	4.5 ± 0.3	15.6 ± 0.6 ***	12.4± 0.6 ***^,###^	<0.0001
Fat mass (%)	12.5 ± 0.7	31.7 ± 0.9 ***	27.6 ± 0.9 ***^,###^	<0.0001
Pregnancy outcome	*n* = 24–31	*n* = 30–33	*n* = 20–26	
Gestation length (d)	20 [19, 20]	20 [21, 22]	21 [20.3–21] ***^,##^	<0.0001
Litter size (no pups)	7.0 [6.0–9.0]	6.0 [5.0–7.0] **	6.0 [5.0–7.0] *	0.0015
PN2 weight (g/pup)	1.9 ± 0.04	1.6 ± 0.04 ****	1.7 ± 0.04 ***	<0.0001

**Table 2:** Primer sequences for RT-PCR.

Gene	Forward 5′-3′	Reverse 5′-3′
*Cd11c*	TGCTGTTGGGGTTTGTTTCTTG	CGAACTCAGCACCGTCCAT
*F4/80*	CACTTCCAAGATGGGTTAACATCC	CTGCCATCAACTCATGATACCCT
*Ppia*	GTCCAGGAATGGCAAGACCA	GGGTAAATGCCCGCAAGTC

## Data Availability

As the manuscript does not include any omics data, the data presented in this study are available on request from the corresponding author as appropriate (such as for use in meta-analyses) following publication. The data are not publicly available.
